# Control of Interface Migration in Nonequilibrium Crystallization of Li_2_SiO_3_ from Li_2_O–SiO_2_ Melt by Spatiotemporal Temperature and Concentration Fields

**DOI:** 10.1021/acsomega.4c02361

**Published:** 2024-05-02

**Authors:** Sanchita Chakrabarty, Haojie Li, Michael Fischlschweiger

**Affiliations:** Chair of Technical Thermodynamics and Energy Efficient Material Treatment, Institute of Energy Process Engineering and Fuel Technology, Clausthal University of Technology, Agricolastraße 4, 38678 Clausthal-Zellerfeld, Germany

## Abstract

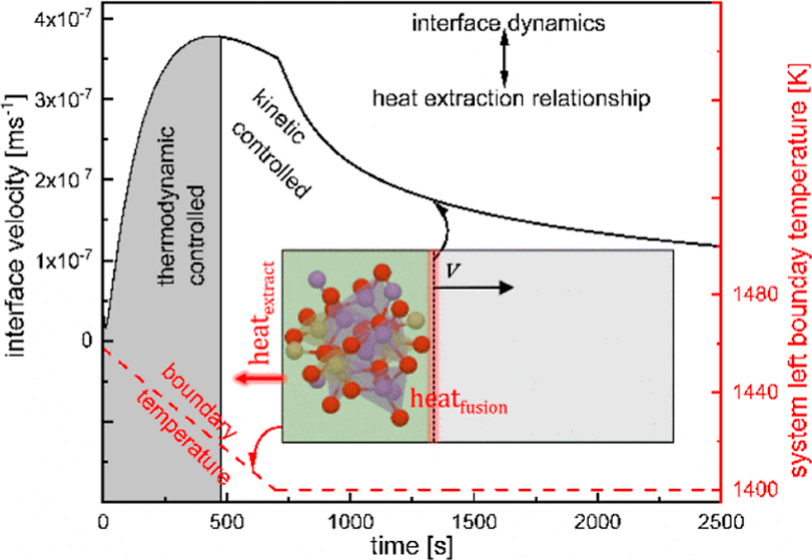

During liquid–solid transformation, bulk mass and thermal diffusion, along with the evolved interfacial latent heat, work in tandem to generate interfacial thermodynamic and kinetic forces, the interplay of which decides the solidification velocity and consequently the solidified phase attributes. Hence, access to interface dynamics information in dependence of bulk transfer processes is pivotal to tailor the desired quantity of solid phases of unique compositions. It finds particular application for engineering concentrated Lithium (Li) phases out of Li-ion battery slags, thus generating a high value-added product from a conventional waste process stream. However, considerable challenge exists to predict the impact of the diverse external cooling rates on the evolving internal transfer processes and thus tuning solidification routes for achieving phases of interest. Hence, in this work, a thermodynamically consistent nonequilibrium model, by considering spatiotemporal temperature and concentration fields, is developed and applied to study solidification of Li_2_SiO_3_ from a Li_2_O–SiO_2_ melt that constitutes an important subsystem of the Li containing battery-recycling slags. The approach treats the sharp solid/liquid interface as a moving heat source. In the presence of different heat extraction profiles, it evaluates the spatial temperature heterogeneity and its implicit correlation to internal material fluxes resulting from maximization of dissipation and consequently the interrelation to interface velocities. Model calculations revealed that irrespective of the external cooling rate, for an initial short time duration, the magnitude of which increased with decreasing cooling rates, the interface velocities show a reducing trajectory directly relatable to the reducing thermodynamic forces due to localized interfacial temperature rise from the generated latent heat of fusion from the initial solidification. This is followed by a thermodynamically controlled regime, whereby for each cooling rate, the interface velocities increase until a maxima, the magnitude of which decreases with decreasing cooling rates. Finally, the interface propagation speeds decrease as controlled by the kinetic regime.

## 1 Introduction

Solidification phase transitions and their associated heat transfer phenomenon are an integral part of a wide range of technical processes. In materials processing, operations such as casting, rapid solidification, additive manufacturing, among others, rely on the interrelation of process parameters with heat and mass diffusion to influence phase change kinetics and engineer solids with special microstructures and desired compositions, which impart novel properties to the materials.^1−3^ For biomedical applications, the cryopreservation and cryosurgery technologies have been advanced based on extensive research on the interplay of thermal gradients and ice formation during freezing of biological tissues.^4^ Heat transfer analysis is imperative for the ongoing development of phase change materials that have generated huge interest in the scientific community as potential energy storage devices to combat the perils of renewable fuels.^5^ Oxide melt solidification has been traditionally investigated in dependence of external thermal profiles and internal heat and material transfer phenomena that finds extensive application in glass and ceramics manufacturing.^6,7^ It is also extremely relevant for refractory freeze-lining formation in pyrometallurgical processes.^8^ However, dearth of similar studies for the application of oxide containing melt as a potential source of element recovery and engineering artificial minerals (EnAM)^9^ poses a hindrance to circular economy solutions, for instance, reintegrating Li from spent Li-ion battery processed slags (oxide melts).^10^ Literature on investigating process conditions for blast furnace slag to form perovskite as a potential source of Ti recovery is present in ref (11). However, such a study has been mostly performed under isothermal conditions. With the increasing focus on sustainability and material recycling, in depth solidification characterization for oxide melts in terms of the interrelation of the external and internal kinetics is important^12^ in order to tailor solid phases with concentrated critical/rare elements and thus utilize it as a high value product.^13^

The key aspect that can be gathered from the above discussion is that, irrespective of the field of application, the understanding of the progress of solidification requires an investigation of the coupled mass and thermal transport mechanisms and its implicit relation to the phase change heat evolution. This enables correlating bulk kinetics for the evaluation of solid/liquid interface conditions that are the decisive factor for the evolution of the solidified phase both structurally and compositionally^14,15^ and hence pivotal to achieve the targeted solidification sequence or the final product.

A broad range of industry relevant variables, such as the melt composition, thermophysical properties, external cooling rate, and others, need to be considered for determining the thermal profile and consequently material profile evolution in the melt that determine the internal kinetics and ultimately the properties, of the solidified product. The extensive experimental investigations of the corresponding influences of the process parameters on solidification characteristics that are found in literature, despite providing the foundation for solidification studies, are resource-intensive.^16,17^ Hence, to comprehend the full spectrum of process-property relationship, a modeling framework that is capable of capturing this phenomenon is of utmost importance.

Existing solidification models might be classified into two broad categories: (i) equilibrium models and (ii) nonequilibrium models. For the former class of models, thermal, chemical, and mechanical equilibrium prevails throughout the system undergoing a phase change. Hence, no influence of the thermal profile or any other internal and external kinetics is considered for solidification. The CALPHAD (calculation of phase diagrams) modeling methodology has been one of the most widely adopted frameworks for equilibrium-based phase formation studies. It focuses on formulating Gibbs energies for different phases and based on the global Gibbs energy minimization subject to certain constraints it identifies the most stable phases under a given condition of temperature, pressure, and composition of the system.^18−20^ From the context of solidification investigation under realistic process conditions, which forces the solidifying system particularly far from equilibrium, CALPHAD provides the essential Gibbs energies for different phases that are imperative to calculate the driving forces existing for nonequilibrium processes and thus for the development of the second category of solidification models. A more comprehensive explanation is given in the Materials, Method, and Theory section. However, it might be mentioned here that the prevalence of diffusion kinetics and/or kinetic barriers for liquid/solid interfacial transformations are not taken into consideration within such a model. Hence, CALPHAD model predictions of solid phase fractions might vary from experimental observations dependent on the real kinetic behavior of the phase transition.^21^

The nonequilibrium models can further be classified based on the kinetics that are captured. Some of them are developed from the mass diffusion viewpoint and consider homogeneous spatial temperature fields.^22^ One such approach is the Scheil Gulliver that considers no solid diffusion and infinite liquid diffusivity.^23^ This might be restricted in terms of describing phase transformations for melt having limited diffusivity.^24^ Other modeling frameworks consider the coupled kinetics of heat and mass transfer with the assumption of an equilibrium at the solid/liquid interface.^25,26^ This approach is restricted to processes without steep thermal profiles, which usually induces nonequilibrium interface conditions. The growth of rapid solidification technology (RST) motivated the evolution of models that can incorporate out of equilibrium behavior at the solid/liquid interfaces that are extremely relevant for rapid interface propagation, which does not allow sufficient time for diffusion and consequently for the interface to reach equilibrium.^27^ Other than RSTs, such models might also be relevant for processes which, even though they do not encounter extreme heating or cooling rates, they have such sluggish material kinetics that it hinders the interface to reach equilibrium. However, for these models, usually the interface conditions in terms of temperature, composition, and velocity are governed by relations derived from phenomenological theories. While the application of such models has been extremely successful in describing a wide range of out-of-equilibrium phase transition behaviors,^27^ especially for the alloy systems, it would be worthwhile to attempt developing a modeling framework that on one hand is capable of capturing all the above-mentioned kinetics and on the other constitutes thermodynamically consistent equations.^28^

The holistic framework developed by Svoboda et al.,^29^ which is based on the thermodynamic extremal principle (TEP) could be considered pivotal for such a formulation. This might help navigating the problems that might sometimes arise during solving phenomenological equations with such a degree of idealization that it essentially misses the physical nature of the formulation and leads to higher number of model parameters.

In TEP,^29^ the system under observation is characterized by certain kinetic parameters. In the context of solidification, the kinetic parameters are the material flux to and from the interface and the interface velocity. Such parameters are estimated by solving evolution equations, which are systematically developed based on the maximization of the entropy production rate principle, and thus, the system evolution can be mapped over time. This framework has been very successfully implemented in the past for alloy systems to describe nonequilibrium phenomena of grain coarsening, evolution of precipitate microstructure, solute driven melting/solidification, rapid solidification of stoichiometric and intermetallic compounds, to name a few.^30−34^ However, for most of the application cases, the homogeneity of the temperature field, both in space and time, was maintained. In our previous works,^35,36^ the original isothermal framework from Svoboda et al.^29^ has been extended to consider external temperature profiles with respect to time and nonisothermal phase evolution were successfully simulated for a solid–solid transformation in the MgO–Al_2_O_3_ system and liquid to solid transformation in the Li_2_O–SiO_2_ system.

In this work, a nonequilibrium model is developed, which considers spatiotemporal temperature and concentration fields and hence allows the control of interface migration in nonequilibrium crystallization. The new approach is applied to the oxidic system Li_2_O–SiO_2_ since it forms an important subsystem of the multicomponent lithium containing slag that is increasingly generated from Li-ion battery processing industries. Such a slag system has garnered much interest among the scientific community as a potential source for Li recycling.^37,38^ However, the interplay of complex kinetics influenced by varied process conditions hinders, to some extent, controlled solidification evolution of the melt system of interest or in other words achieving the desired element recovery. Engineering particular crystals out of the slag with a high concentration of the element of interest can only be performed when the internal process kinetics can be understood and tailored according to external process parameters in terms of heat extraction profiles. In this work, the application of the TEP theory elucidates the variation of the local internal kinetics and its effect on the propagation of the solid/liquid interface, in dependence of the tunable external thermal dynamics. Hence, this work could be an initial step toward investigating governing process kinetics prevailing during a multicomponent oxide melt solidification with a new principle aim of triggering preferential out-of-equilibrium crystal phases through controlled solidification strategies.^39,40^

## 2 Materials, Method, and Theory

In this work, the mixed kinetics of heat transfer, mass diffusion, and interface migration are employed to a Li_2_O–SiO_2_ melt for evaluating a 1D crystallization evolution of Li_2_SiO_3_ phase. The nature of solid/liquid interface is considered to be sharp in accordance with the literature investigations for oxidic systems.^41^ The theoretical background for the modeling framework is presented here in three parts. First, the physical model is described such that the governing kinetics are introduced. Next, the isothermal TEP evolution equations are described. Finally, the methodology for extending such a model to incorporate spatiotemporal temperature gradients is presented and discussed.

In this work, a bulk composition of 45.2 mol % Li_2_O and 54.8 mol % SiO_2_ that on equilibrium cooling generates Li_2_SiO_3_ crystals^42^ has been chosen for investigating the spatiotemporal temperature and concentration fields and correlating them with heat evolution during the phase transition. The representative composition and temperature range have been presented in the Supporting Information in Figure S1. Kinetic melt cooling experiments have already been performed for such a concentration in our previous work,^36^ and kinetic parameters (discussed in detail in the following section) required for TEP modeling have already been estimated.

For the progression of solidification, an already nucleated solid/liquid interface is assumed in this work, and consequently the solidification kinetics is studied with respect to the movement of the existing interface that is not influenced by further nucleation. For the crystallization of a solid of concentration 50 mol % Li_2_O and 50 mol % SiO_2_ from a melt of higher SiO_2_ concentration, the following processes must take place simultaneously:^43^

(i) Incorporation of the Li_2_O and SiO_2_ from melt to the solid at the interface, which means interface migration.
(ii) Evolution of latent heat during solidification at the interface.
(iii) Diffusion of the excess SiO_2_ rejected at the growing interface to the melt side into the bulk melt and simultaneous diffusion of Li_2_O from the bulk toward the interface.

The interface migration needs to be assumed infinitely fast for equilibrium conditions to exist at the interface. However, in this work, such assumption can be relaxed and a finite mobility exists for the interface, which signifies nonequilibrium conditions. The interface also acts as a heat source as the solidification is associated with latent heat generation. Thus, it influences the evolution of the bulk temperature gradient, which in turn is implicitly coupled to processes i and iii.

**Figure 1:**
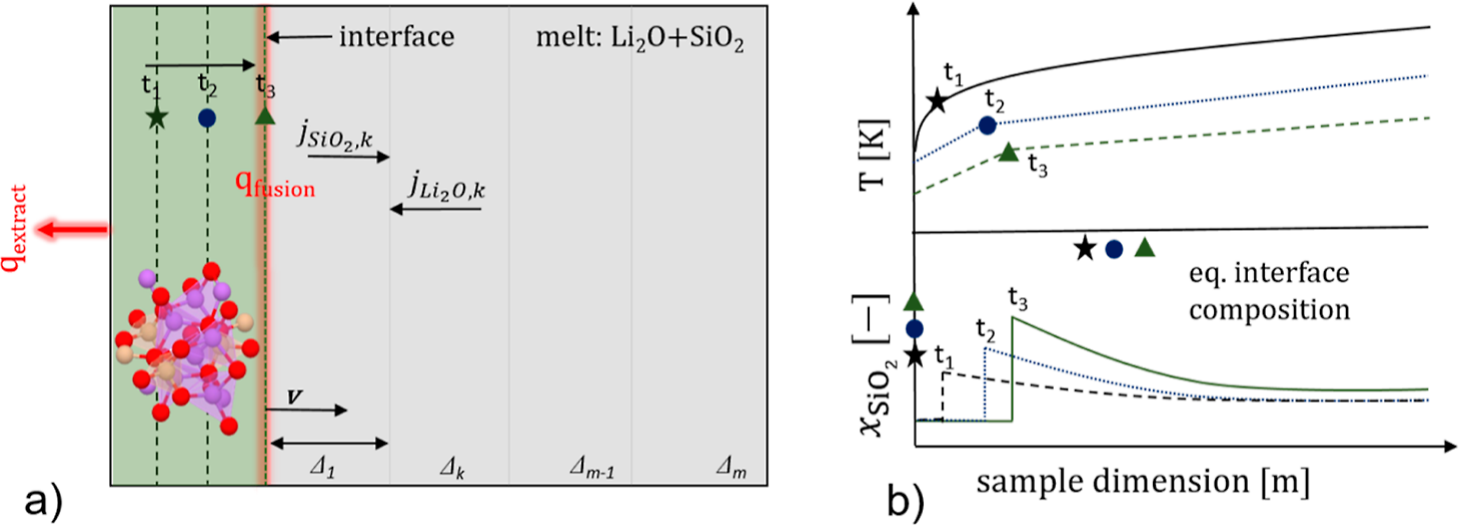
(a) Schematic representation of 1D Li_2_SiO_3_ crystallization along with the allocation of *q*_fusion_ at the interface; the representative solid structure is computed based on ref (44). (b) Heterogeneous temperature and composition field evolution over time; the star, circle, and triangle symbols on the temperature profiles represent interface temperatures; the same on the *y* axis representing mole fraction show equilibrium mole fractions at respective interface temperatures.

A schematic drawing of the system is presented in Figure 1a. The system is spatially discretized into regions of thickness Δ_*k*_ (*k* being the number of discretization). When the system is subjected to a certain heat extraction profile, *q*_extract_ at one boundary, the existing interface starts to migrate with a velocity *v* into the melt and a generation of the latent heat of fusion *q*_fusion_. For a positive *v*, the interface acts as a moving heat source due to the continuous evolution of the latent heat of solidification. Three time instances, *t*_1_, *t*_2_, and *t*_3_, along with the respective interface positions have been presented by the black star, blue circle, and green triangle. The development of the temperature field with time within the system is depicted in Figure 1b. During solidification, each region experiences a net positive flux of SiO_2_, *j*_*i,k*_ (*i* = SiO_2_) and a net negative flux of Li_2_O. The enrichment of SiO_2_ at the interface with time is also presented in Figure 1b. The difference between the actual interface concentration and the equilibrium concentration for the particular temperature existing at the interface has been presented for the three time instances, *t*_1_, *t*_2_, and *t*_3_.

**Figure 2:**
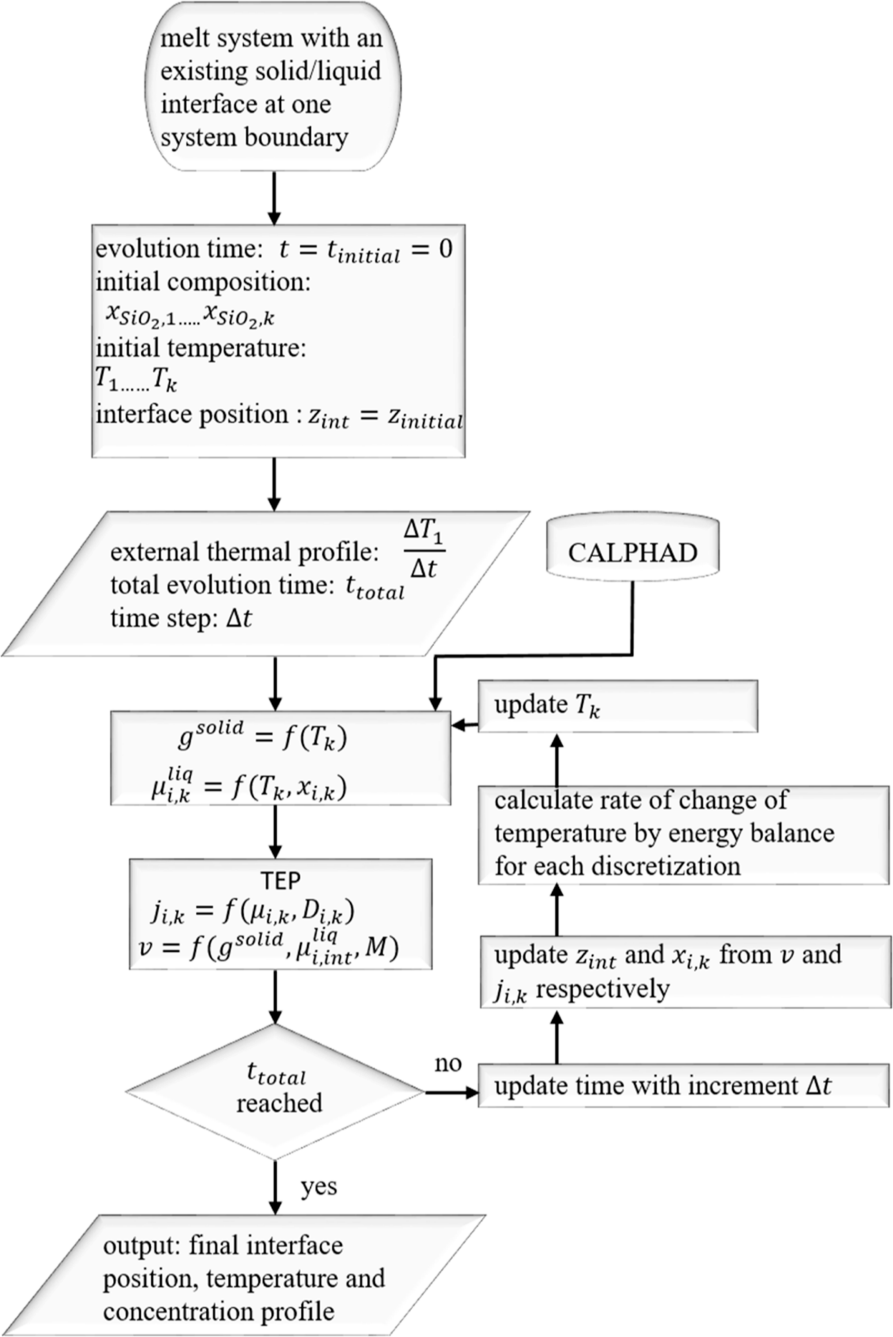
Flowchart for solving a liquid–solid transition problem with the TEP framework considering a spatiotemporal temperature field. Further details on the solution of TEP equations are provided in the Supporting Information.

The flowchart for solving the system evolution under certain given external conditions is presented in Figure 2. To start the process, a system that consists of a Li_2_O–SiO_2_ containing melt, along with a solid section of Li_2_SiO_3_ on its left boundary, is considered. A homogeneous distribution of composition (within the melt) and temperature for the initial time (*t*_initial_ = 0) is given. An external temperature profile and the total evolution time are given as input to the process. Based on these conditions, to track the overall solidification kinetics, first, the Gibbs energy, *g*^solid^ of the crystalline Li_2_SiO_3_ and the chemical potentials μ_*ik*_ for each component, (*i*: Li_2_O, SiO_2_) in each discretization (*k*) of the melt, for the respective temperature, *T*_*k*_ and composition, *x*_*ik*_ given for time *t*, are directly called from the CALPHAD database^42^ via ChemAPP. A more detailed explanation of the principle coupling procedure is given in ref (35).

The CALPHAD information is required to solve the TEP evolution equations in the next step. Mass flux and interface migration are some of the kinetic processes that dissipate the Gibbs energy during solidification. First, the change rate of the total Gibbs energy, *Ġ* is expressed in terms of the kinetic parameters *j*_*ik*_ and *v* that represent the kinetic processes. Next, the dissipation function *Q*, which is a linear combination of fluxes and forces (according to the TEP), is formulated. Finally, the principle of maximum entropy production principle is implemented by carrying out a constrained maximization of *Q* with respect to the kinetic parameters. The mathematic correlation thus derived between *Ġ* and *Q* can be solved to generate the kinetic parameter values over time and map the system evolution. For an isothermal system, within the TEP framework Svoboda et al.,^29,45^ derived the rate of Gibbs energy, *Ġ*, in terms *j*_*ik*_ and *v* that represent the kinetic processes of mass flux (in liquid) and interface migration, respectively. These are presented in eqs 1 and 2: For a detailed derivation of the expressions the readers might refer to ref. (45).
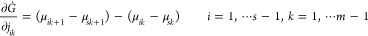
1

In eq 1, *s* is the number of components and *m*, the number of discretization.
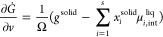
2

In eq 2, the subscript “int” represents a value at the first discretization on the liquid side “liq” of the interface. Ω is the partial molar volume of the components.

In addition to *Ġ*, the information for the partial change of the total dissipation required to solve the system evolution in terms of the kinetic parameters are represented in eqs 3 and 4.^45^
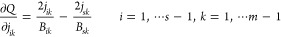
3

In eq 3, *Q* is the total dissipation, ; , *x* and *D* are mole fraction and diffusion coefficient of the components in liquid, respectively. *R* is the universal gas constant and *T* is the system temperature. Here, it should be mentioned that for the first time step, the temperature distribution is considered homogeneous throughout the system. Equation 4 reads
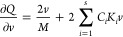
4

*C*_*i*_ = *K*_*i*_*U*_*i*,int_; ; *M* is the solid/liquid interface mobility coefficient.

The kinetic parameters *D* and *M*, stated in eqs 3 and 4, are assumed to follow Arrhenius law as is given in eq 5.^46^
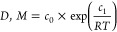
5

For the Li_2_O–SiO_2_ system, the pre-exponential and the exponential coefficients presented in eq 5 are inversely estimated from a set of experimental data on phase evolution for the same system reported in our previous work.^36^ The values are presented in Table 1.

**Table 1:** Parameters for Representing Kinetic Coefficients as Functions of Temperature

parameters	*D*_Li_2_O_ [m^2^ s^–1^]	*D*_SiO_2__ [m^2^ s^–1^]	*M*_solid/liquid_ [m^4^ J^–1^ s^–1^]
*c*_1_ [m^2^ K s^–1^](*D*)/[m^4^ K J^–1^ s^–1^](*M*)	–1.75 × 10^5^	–2.21 × 10^5^	–1.4 × 10^5^
*c*_0_ [m^2^ s^–1^](*D*)/[m^4^ J^–1^ s^–1^](*M*)	1.06 × 10^–3^	9.84 × 10^–4^	4.1 × 10^–7^

Finally, according to the constrained maximization of the rate of entropy production following TEP, eq 6 is given^45^
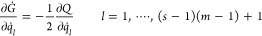
6where *q̇*_*l*_ represents the kinetic parameters for an isothermal system, *j*_*ik*_ and *v*, respectively. Substitution of eqs 1–4 in eq 6 results in a set of linear equations, which need to be solved to obtain the kinetic parameters that describe the system evolution. The substitution and updating the system parameters are further explained in the Supporting Information via eqs S1–S6.

Following the TEP solution step, a decision was made whether to continue the solidification simulation for further time steps. If not, then the system remains at the present state. If yes, the time was updated and simultaneously the composition field and the interface positions are updated from the information on *j*_*ik*_ and *v*, respectively, in accordance with a forward Euler numerical scheme. The detailed procedure is given in Figure 2.

The application of an external boundary temperature profile along with progression of solidification that creates a heat source term at the interface instigates thermal diffusion to occur within the system. The effect of thermal diffusion can be accounted for^47^ fully by solving the isothermal TEP simultaneously with a heat transfer equation. Therefore, for the first time, a spatiotemporal thermal gradient is introduced to describe a liquid–solid transition within the framework of TEP by solving the evolution eq 6 in parallel to a 1D heat conduction equation given as follows^48^
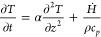
7

Equation 7 represents the change in temperature *T* for a particular discretization of *z* in the system over time *t*. α represents , where *k* is the thermal conductivity of the system, ρ is the density, and *c*_*p*_ is the specific heat capacity. The parameter values of eq 7 are derived from CALPHAD database^42^ and represented in Table 2.

**Table 2:** Thermophysical Parameters of the System

	ρ [kg m^–3^]	*c*_*p*_ [J kg^–1^ K^–1^]	*k* [W m^–1^ K^–1^]
Li_2_O–SiO_2_ melt	2111	1840	0.2
Li_2_SiO_3_ (solid)	2570	1890	8

*Ḣ* in eq 7 is the heat generation rate per volume for each discretization. This value is zero for all discretization except the one that contains the interface where the latent heat of phase change is either released for solidification or absorbed for melting. Therefore, in this framework, the heat source/sink is located at the interface. The total heat generated *q*_fusion_ in each step is calculated based on the interface velocity information, *v*, coming from TEP.

To solve the above partial differential equation for any time step, the information on the temperature profile of the previous time step is inserted as the initial condition. Furthermore, for the left boundary, the rate of change of temperature is determined by the external cooling rate, and the right boundary is considered to be under the adiabatic condition.

The time increment in eq 7 that was implemented in the model was first tested such that the Courant–Friedrichs–Lewy (CFL) condition was satisfied.^49^

Finally, eq 7 was solved by applying the Crank Nicolson method, and the evolution of the temperature profile considering the external cooling rate and internal heat generation was achieved.

Following this step, procedures are executed according to Figure 2. The final temperature and composition fields along with the interface position are then presented as output.

## 3 Results and Discussion

In this section, the modeling framework developed to couple both heat and mass transfer associated with phase transition has been implemented to simulate the crystallization kinetics of Li_2_SiO_3_ from a Li_2_O–SiO_2_ melt. Experimental determination of the same in our previous work^36^ was performed for such a process domain, in terms of system magnitude and external cooling profiles that thermal transport within the system was fast enough in comparison to other process kinetics, to equilibrate the system in terms of temperature at each local time step and hence a homogeneous spatial distribution of temperature could be considered for model calculations. However, the situation changes in the context of slag solidification whereby the existence of extreme boundary thermal profiles and/or the presence of already existing solid phases creates significant heterogeneity in the spatial temperature field. To develop processing strategies in terms of cooling profiles and isothermal retention times in this scenario, for engineering particular crystallites (EnAM) out of the slag, evolution of solid/liquid interface kinetics needs to be investigated in dependence of varying thermophysical properties prevailing within a system due to the coexistence of a solidified state along with the melt that promotes a considerable spatial gradient of temperature. Thus, in this study, solidification velocity for Li_2_SiO_3_ from the Li_2_O–SiO_2_ melt is modeled by considering such a system dimension and initial solid Li_2_SiO_3_ phase fraction that significant spatial temperature heterogeneity can develop within the system for the time interval and implemented external cooling profiles studied. This enables a comparative study, with systems having homogeneous spatial temperature fields. Solid/liquid interface velocities are investigated as a function of the interface driving forces that in turn are studied in dependence on the bulk kinetics generating from varying boundary thermal conditions.

### 3.1 Li_2_SiO_3_ Phase Evolution Investigated for Two Cases:1, 2; for Both the Cases, the Left Boundary of the System Is Cooled at a Rate of 5 K min^–1^ until It Reaches 1400 K; Following This for Case 1, the Left Boundary Temperature Is Held Constant @ 1400 K for 30 min (Profile 1); for Case 2, the Left Boundary Is Considered Adiabatic (Profile 2)

For the model simulation, a Li_2_O–SiO_2_ melt of composition 45.8 mol % Li_2_O and 54.2 mol % SiO_2_ at a temperature of 1458 K is considered. Initially, the melt is homogeneous, with respect to temperature and composition. An initial fraction of solid stoichiometric compound Li_2_SiO_3_ is considered to coexist with the melt at the left boundary of the system at the same temperature. At first, a cooling rate of 5 K min^–1^ is applied at the system left boundary until the temperature reaches 1400 K. Two thermal profiles, namely, profiles 1 and 2 are implemented thereafter. For profile 1, the temperature on the left boundary is maintained at 1400 K for 30 min. For profile 2, an adiabatic condition is applied for 30 min. The right boundary is subjected to an adiabatic condition throughout the process. For clarity, the entirety of the process that includes cooling and profile 1 would be referred to as case 1 and the process including cooling and profile 2 as case 2 hereafter.

**Figure 3:**
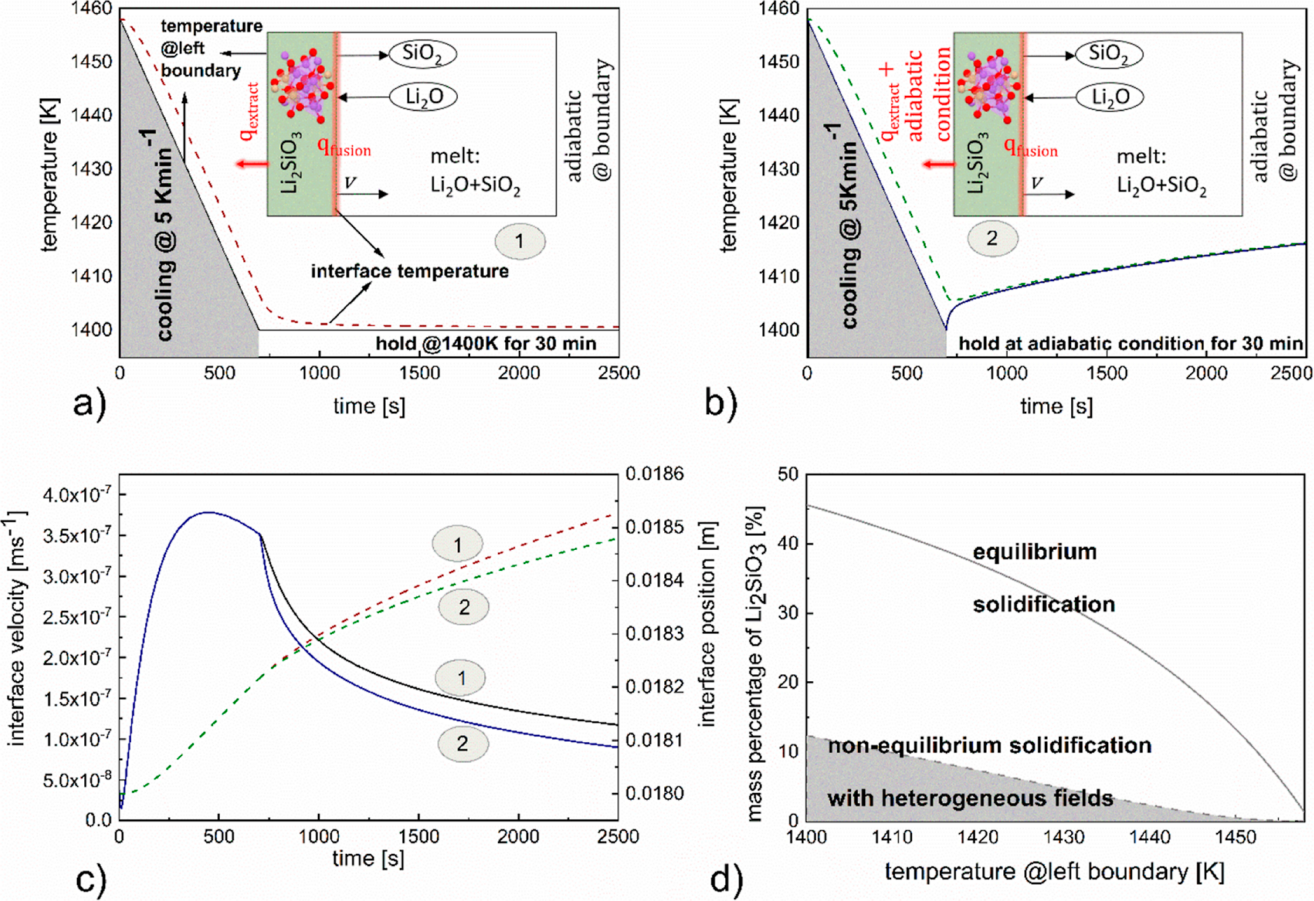
(a) Left boundary temperature and interface temperature represented by the black solid line and brown dashed line, respectively, for case 1. (b) Left boundary temperature and interface temperature represented by the blue solid line and green dash line, respectively, for case 2. (c) Interface velocity and position represented by the black solid and brown dash lines, respectively, for case 1 and blue solid and green dash lines, respectively, for case 2. (d) Mass fraction of solid Li_2_SiO_3_ as a function of left boundary temperature denoted by the gray dash curve; the gray region indicates the continuous cooling period, and the corresponding equilibrium mass percentage is denoted by the gray solid line.

For both the model cases, which have been schematically represented as 1 and 2 in Figure 3a,b, migration of the solid/liquid interface into the melt has been observed and the corresponding interface positions have been represented in Figure 3c by the brown dashed line and green dashed line for the first and second cases, respectively. A positive interface displacement with time corresponds to the crystallization of Li_2_SiO_3_ from the liquid melt and is associated with the evolution of the latent heat of solidification at the interface. Such phenomena coupled with the external heat extraction profile create a spatial temperature gradient. To depict the same, the variation of the left boundary temperature and the interface temperature over time have been represented by the black solid line and brown dashed line, respectively, for case 1 and the blue solid line and green dashed line, respectively, for case 2 in Figure 3a,b respectively.

It can be observed from Figure 3a,b by comparing the corresponding profiles for the two model cases that, as long as the left boundary was subjected to a continuous cooling rate, the interface temperature along with the left boundary temperature showed identical decreasing trajectories.

Following this, when the constant boundary temperature condition was implemented for case 1, the interface temperature continued to decrease, albeit with a reduced slope. In spite of the discontinuity of the boundary cooling rate, which in effect correlates to less heat being extracted from the system, the decreasing trend of the interface temperature could be attributed to the fact that during this period, the solidification velocity, as has been depicted by the black solid line in Figure 3c, also showed a decreasing profile that can be correlated directly to a reducing heat generation rate within the system.

On the contrary, the model captured an increasing trend for both the left boundary and interface temperature, as can be observed from Figure 3b when the adiabatic condition was implemented at the left boundary. Although the solidification velocity in this case (case 2) as has been represented by the solid blue line in Figure 3c, and hence the heat generation rate, is less than case 1, since no heat flux out of the system is present, the melt heat from the higher temperature region (bulk melt in the vicinity of right boundary) that flows toward the left boundary, equilibrates and with time raises the respective temperatures at the left boundary and interface.

The evolution of the interface velocity, a key determinant of the characteristics of the phase that solidifies out of the melt, over time, and its variation in dependence of the heat extraction profiles is further analyzed from Figure 3c. As can be observed that the period during which the left boundary was subjected to continuous cooling @ 5 K min^–1^, the interface velocity (identical for both cases) increased until 3.78 × 10^–7^ ms^–1^ as the interface temperature is cooled from 1458 to ∼1429 K. Beyond this point, the velocity starts decreasing and continues this trend for both the cases even when the different boundary thermal profiles (profile 1 and 2) are applied. However, the slope of the decreasing velocity is steeper for case 2.

Figure 3d represents a comparison between the mass percentage of Li_2_SiO_3_ solidified under equilibrium conditions based on CALPHAD database^42^ to the calculated nonequilibrium solid percentage under the continuous cooling path of 5 K min^–1^. This could be considered as an upper bound for the solidification mass percentage.

To investigate the nature of the velocity profiles, the influencing factors, which are the thermodynamic driving force |Δ*g*| = *g*^*solid*^ – (*x*_SiO_2__^solid^ μ_SiO_2__,_int_^liq^ + (1 – *x*_SiO_2__^solid^ μ_Li_2___O,int_^liq^) (as can be observed from eqs 2 and 4), and the kinetic forces related to the diffusion and interface mobilities (as can be observed from eqs 1–4) are further analyzed. The analysis is subdivided into the following intervals:

#### 3.1.1 Continuous Cooling @ 5 K min^–1^ at the Left Boundary

**Figure 4:**
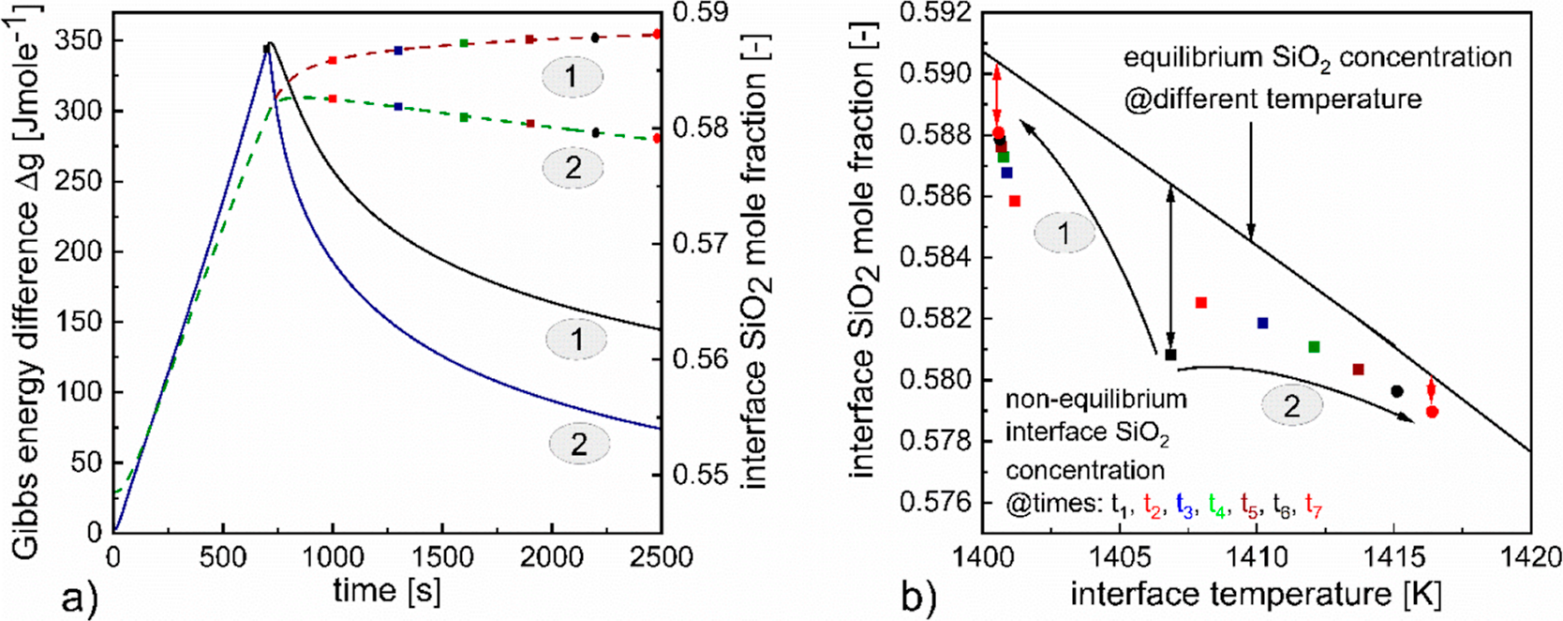
(a) |Δ*g*| over time represented by the black solid line for case 1 and blue solid line for case 2; interface SiO_2_ mole fraction variation over time represented by the brown dashed line for case 1 and green dashed line for case 2. (b) Equilibrium melt SiO_2_ mole fraction variation with temperature presented by the black solid line; the black, red, blue, green, brown squares and black and red circles represent the actual interface temperature and composition that exists in the system for the respective times denoted in (a) with similar shapes.

The thermodynamic force prevailing at the interface (identical for cases 1 and 2) and represented by the solid black and blue lines in Figure 4a is found to increase with the decreasing interface temperature. On the other hand, the kinetic forces decrease with temperature. Considering the competing effect of the influencing forces, the increasing interface velocity until ∼1429 K might indicate the dominance of the thermodynamic force. Lowering the interface temperature further, the observed decrease in velocity despite the increasing thermodynamic force might indicate a kinetic controlled growth.

#### 3.1.2 Application of Profiles 1 and 2 at the Left Boundary

For case 1, when the temperature of the left boundary is held constant, it can be observed from Figure 4a that |Δ*g*| shows a decreasing trend. Additionally, the kinetic forces continue to decrease, corresponding to the decreasing interface temperature. Hence, both the influencing factors contribute to the decreasing interface velocity represented in Figure 3c.

For case 2, when the adiabatic left boundary condition is implemented, on one hand, |Δ*g*| showed a decreasing profile similar to case 1 but with a steeper slope. On the other, the kinetic forces increased with the increasing interface temperature as is evident from Figure 3b. The decreasing trend of the interface velocity in this case as is represented in Figure 3c indicates a shift to the thermodynamic controlled regime from the kinetic controlled one that was prevalent in (3.1.1).

The variation of |Δ*g*| in dependence of the external heat extraction profiles, as has been mentioned in above discussion (3.1.2) and represented in Figure 4a, is further investigated to find its correlation with processing relevant factors, namely, interface composition and temperature. Such an analysis could elucidate the implicit relation between internal process dynamics and the process parameters that are, in turn, influenced by external operating conditions. The discussion for |Δ*g*| hereafter is done for the interval when different thermal profiles have been applied to case 1 and 2.

The interface conditions change in terms of temperature, as depicted in Figure 3a,b, along with composition, as depicted by the brown dashed line and green dashed line for cases 1 and 2 in Figure 4a. A direct relation between the interface composition with |Δ*g*| is not observed.

To demonstrate the interrelation between temperature, composition, and |Δ*g*|, for case 1, the information of the interface compositions at seven time instances, namely, 696, 996, 1296, 1596, 1896, 2196, and 2496 s represented as black square, red square, blue square, green square, brown square, black circle, and red circle in Figure 4a, are represented corresponding to their temperatures in Figure 4b.

The variation of equilibrium composition with temperature is also presented in Figure 4b. It can be observed that for case 1, the deviation of the interface composition with the equilibrium composition for the corresponding temperature decreased, and this influenced |Δ*g*| for case 1 to decrease over time. From an analogous study for case 2, represented in Figure 4b, it can be observed that while the interface temperature increased with time, the interface composition first showed an increasing trend, followed by a decreasing trend. However, irrespective of the increment or decrement of the individual values of interface composition and temperature, the deviation of the interface composition from the equilibrium composition for the corresponding temperature decreased over time, as in case 1, causing a reduction in the |Δ*g*| value. Comparing between cases 1 and 2, a steeper reduction for the |Δ*g*| value for case 2 is observed in Figure 4a. This might be explained following the above argument that at each time instant, a greater deviation is observed for case 1 in Figure 4b compared to case 2. This has been exemplified for the time instant 2496 s with the red double headed arrow, where the difference is most pronounced.

### 3.2 Li_2_SiO_3_ Phase Evolution for Different Cooling Rates of 1.5, 5, and 12 K min^–1^: Comparison between the Systems, with and without a Spatial Temperature Gradient

**Figure 5:**
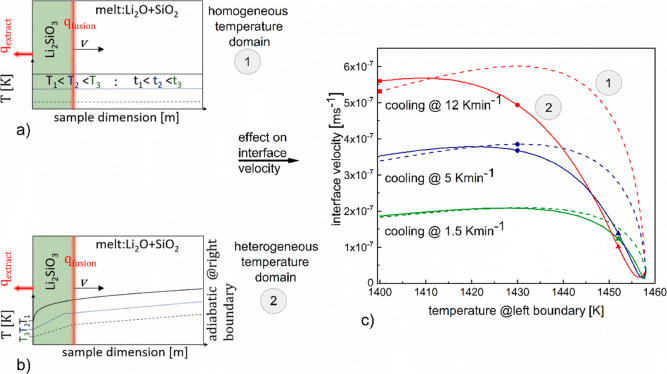
(a) Schematic representation of liquid to solid transformation with interface heat generation in a system with homogeneous temperature profile (case 1). (b) Schematic representation of liquid to solid transformation with interface heat generation in a system with heterogeneous temperature profile (case 2). (c) Interface velocity with respect to system left boundary temperature for different cooling rates are shown: cooling rates 1.5, 5, and 12 K min^–1^ are presented by green, blue, and red colors, respectively. Case 1 is represented by the dash lines, and case 2 is represented by the solid lines; triangle, circle, and square represent the instances when left boundary temperature reaches 1452, 1430, and 1400 K respectively.

The Li_2_O–SiO_2_ melt system coexisting with an initial solid fraction of Li_2_SiO_3_, at 1458 K, is subjected to three different cooling rates of 1.5, 5, and 12 K min^–1^. For each cooling rate, two cases have been simulated, as represented schematically in Figure 5a,b. For case 1, at each time interval, the system is assumed to be spatially homogeneous with respect to temperature, i.e., at any time interval, when the system is cooled from a higher to the lower temperature, the whole system is assumed to achieve the lower temperature. Such a scenario might arise when for the same cooling rates, heat flux outside the system occurs from all boundaries and the latent heat generated is not sufficient enough to create a spatial temperature gradient. For case 2, the spatial gradient for temperature forms within the system, i.e., cooling rate is applied only at the left boundary and temperature for the rest of the system evolved due to 1D heat conduction considering the latent heat generation due to solidification at the interface. Thus, for a particular cooling rate, although the system experienced the same temperature condition at the boundary (left) for both case 1 and case 2 for any particular time, the effect of the internal energy transfer prevailing for case 2 subjected the solid/liquid interface for this case to experience different conditions of temperature and composition compared to case 1 for the same time instance. The resulting variation in interface velocities for the two cases investigated for the above-mentioned cooling rates is represented corresponding to the system boundary temperature in Figure 5c. In the figure, the profiles denoted by green, blue, and red colors represent the cooling rates 1.5, 5, and 12 K min^–1^ respectively. The corresponding dashed and solid lines depict model calculations for case 1 and case 2.

As can be observed from Figure 5c, when the system boundary is cooled from 1458 to 1400 K, the interface velocities for each cooling rate, for case 1, shows an increasing profile until a particular temperature followed by a decreasing trend. While this holds true also for case 2 for the majority of the investigated simulation interval, an initial decreasing profile, before it starts to increase is observed for a short interval in this case. This is most pronounced for the highest cooling rate, 12 K min^–1^.

Comparing the three cooling rates for case 1, for any system at the left boundary temperature, the interface velocity is the highest for the highest cooling rate of 12 K min^–1^ followed by the decreasing cooling rates of 5 and 1.5 K min^–1^ respectively. Excluding the temperature interval between 1458 and ∼1446 K, a similar trend for the velocity profiles for each cooling rate is observed for case 2. It can further be observed that for each cooling rate, an initial temperature interval exists for which the interface velocity for case 1 is higher than for case 2. Beyond this interval, the reverse is observed. The interval increases with an increasing cooling rate.

**Figure 6:**
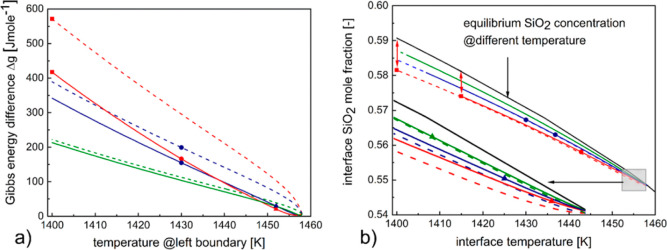
(a) |Δ*g*| with respect to system left boundary temperature for case 1 and case 2 (represented by dashed and solid lines) for the three cooling rates 1.5, 5, and 12 K min^–1^ are presented by green, blue, and red colors, respectively. (b) Corresponding interface temperature and composition that exists in the system. The equilibrium melt SiO_2_ mole fraction variation with temperature is presented by a black solid line.

To investigate the influencing factors for the above observed variation in interface velocities in dependence of external heat extraction profiles (different cooling rates) and internal process dynamics (existence or absence of spatial temperature gradient), three instances, denoted by the triangle, circle, and square, when the system left boundary temperature reaches 1452, 1430, and 1400 K, respectively, are analyzed further. The corresponding colors for each shape refers to the different cooling rates. Analogous profile outline (solid, dashed), colors (green, blue, red) and shapes (square, circle, triangle) are used to represent the variation of |Δ*g*| over system boundary temperature and the interface composition vs interface temperature profiles in Figure 6a,b respectively. The interrelation between interface temperature, composition, |Δ*g*|, and the interface velocity is demonstrated for the three instances as follows:

#### 3.2.1 Comparing Interface Velocities for Cases 1 and 2 for the Cooling Rate 12 K min^–1^ When the Left Boundary Temperature for Each Case Reached 1400 K

The interface velocity for case 2 as is observed from Figure 5c is comparatively higher than case 1, in spite of a higher thermodynamic driving force |Δ*g*| prevailing at the interface for case 1, which has been represented in Figure 6a. This higher value of |Δ*g*| for case 1 can be justified from the fact that at its corresponding interface temperature (same as left boundary temperature), the deviation of the interface composition from the equilibrium composition, as is represented by the red double headed arrow in Figure 6b is higher than that for case 2 at its respective interface temperature. It is noteworthy to mention here that due to the existence of a heterogeneous temperature field for case 2, the interface temperature for this case is higher than the left boundary temperature that has reached 1400 K. This can also be observed from Figure 6b. Hence, the interface kinetics for case 2, which is at a higher temperature ∼1414 K, is greater than that for case 1, the interface temperature for which is at 1400 K. This correlates to a higher kinetic force for case 2 that compensates for the higher thermodynamic force existing for case 1 and results in a faster interface propagation speed for case 2.

#### 3.2.2 Comparing Interface Velocities for Cases 1 and 2 for the Cooling Rate 5 K min^–1^ and for Case 2 for the Cooling Rate 12 K min^–1^ When the Left Boundary Temperature for Each Case Reached 1430 K

The higher interface velocity for case 2 of the cooling rate 12 K min^–1^ compared to case 1 of the cooling rate 5 K min^–1^ as is observed from Figure 5c in spite of a higher |Δ*g*| for the latter as observed from Figure 6a might be explained following the above argument that the existent higher kinetics for the former, due to the prevailing higher temperature condition, compensates for the higher |Δ*g*| of the latter.

However, comparing the influencing forces for cases 1 and 2 of the cooling rate 5 K min^–1^, it is observed that although a higher kinetic force exists at the interface for case 2, due to the higher temperature condition, it is not enough to compensate for the higher thermodynamic force available for case 1, and hence, the interface velocity for case 2 is lower.

#### 3.2.3 Comparing Interface Velocities for the Cooling Rates 1.5, 5, and 12 K min^–1^ for Case 2 When the Left Boundary Temperature for Each Case Reached 1452 K

The competition between the governing kinetic and thermodynamic forces to determine the interface velocities is further exemplified for this instance where the interface velocity, as observed from Figure 5c, is the highest for 5 K min^–1^ since, on the one hand, the kinetic forces trump over the thermodynamic force when compared to the condition for the cooling rate 1.5 K min^–1^ while on the other a higher thermodynamic force for 5 K min^–1^ compared to that existent for 12 K min^–1^ makes the interface velocity faster for the former.

It might also be interesting to observe that for each cooling rate, at the initiation of the simulation interval, for a short duration, the interface velocities present a decreasing trend. This might be directly correlated to the reducing thermodynamic forces due to localized interfacial temporal temperature rise from the generated latent heat of fusion during the initial solidification. However, with time, the heat extracted compensates for the generated heat and the interface experiences a temporal temperature drop as is presented in Figure 6b.

Following the above discussion, it might be noteworthy to mention here that from a processing point of view, to achieve certain desired solidified phase characteristics that are highly dependent on the interface velocities, a fundamental understanding of the prevalent interface driving forces and its correlation to external operating conditions is of utmost importance.

## 4 Conclusions

The mixed kinetics of heat and mass transfer prevalent during melt solidification is captured for the first time using the thermodynamically consistent framework of TEP for controlling interface migration in nonequilibrium crystallization. Allocating the heat of fusion at the moving sharp solid/liquid interface and accounting for the varying internal thermophysical properties due to the coexistence of solid and liquid phases, a heat equation was solved coupled with the TEP evolution equations. In the presence of different boundary conditions in terms of heat extraction profiles, the time evolution of the solid phase formed and thermal profiles within a system are described. Such a framework is essential for the description of the kinetic evolution of the solid/liquid interface in terms of both temperature and composition. This is crucial for understanding the evolving solidifying phase, especially in the vicinity of the interface.

The developed modeling strategy is employed for describing the solidification of Li_2_SiO_3_ from the Li_2_O–SiO_2_. The complex interplay of bulk mass and heat transfer on the generation of interfacial thermodynamic and kinetic forces that propagate solidification, which in turn implicitly influences the mixed kinetic of the system could be revealed through the modeling investigations.

The kinetic forces for interface mobility and bulk diffusion decrease with the temperature. Thus, bulk and interface mass transfer is proportional to the heat transfer that in turn is dependent on the spatial and temporal thermal gradient evolved within the system in the presence of an external heat extraction profile and interface latent heat evolution. Additionally, the thermodynamic forces of the phase transition decrease when the interfacial composition nears the equilibrium composition at the evolved interface temperature. Considering the dual forces, a multitude of temperature–composition conditions arise at the solid/liquid interface, depending on external cooling profiles, whereby, for certain instances, the thermodynamic and kinetic forces act congruently to influence the interface velocity. For other processing cases, however, the forces counteract each other, and depending on their relative magnitude, solidification is either thermodynamic-controlled or kinetic-controlled.

For all the cooling rates investigated, a rapid latent heat evolution from the initial solidification that corresponded to the starting melt conditions was observed to raise the interfacial temperature. This consequently decreased the solidification velocity for a short duration before the boundary heat extraction rates compensate for the generated heat and start reducing the interface temperature. This, in turn, increases the interface velocity. This information is important for coupled microstructure and process design and helps to further understand structure-process-property relationships in nonequilibrium solidification.

## Supporting Information Available

Relevant part of the Li_2_O–SiO_2_ binary phase diagram on which the modeling temperature and composition ranges are based and TEP equations to support the equations provided in the manuscript (PDF)
