# Supplementary material for: Control of Interface Migration in Nonequilibrium Crystallization of Li2SiO3 from Li2O–SiO2 Melt by Spatiotemporal Temperature and Concentration Fields
Source: ACS Omega. 2024 May 2;9(19):21557–68. doi: 10.1021/acsomega.4c02361 (PMC11097345; doi:10.1021/acsomega.4c02361)
Supplement: Supplementary file 1 — ao4c02361_si_001.pdf [file ao4c02361_si_001.pdf]

Supplementary Material

Control of Interface Migration in Nonequilibrium Crystallization of Li_2SiO_3 from Li_2O-SiO_2 Melt by Spatiotemporal Temperature and Concentration Fields

Sanchita Chakrabarty[1], Haojie Li[1], Michael Fischlschweiger[1*]

[1] *Chair of Technical Thermodynamics and Energy Efficient Material Treatment, Institute of Energy Process Engineering and Fuel Technology, Clausthal University of Technology, Agricolastraße 4, 38678 Clausthal-Zellerfeld, Germany*

Relevant part of Li_2O-SiO_2 equilibrium phase diagram

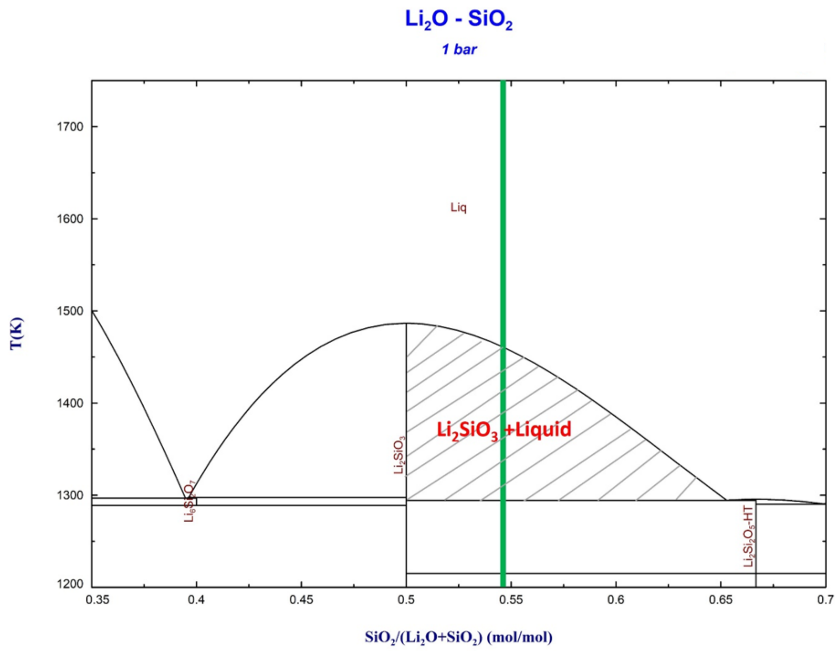

Fig S1: Li_2O-SiO_2 phase diagram [1,2]; the green vertical line represents the starting composition of the melt chosen for model simulations; the temperature range for calculations is always within the two phase region of Li_2SiO_3 and liquid.

TEP equations

The Li_2O-SiO_2 melt, containing 2 components, s=2, is discretized into m discretization of thickness Δ_k. j_{ik} (i=1,2; k=1,..,m-1) and v are solved following the TEP methodology. Substituting equations 1 and 3 in equation 6 from the manuscript, the following equations are derived [3]:

$$(\mu_{ik+1} - \mu_{sk+1}) - (\mu_{ik} - \mu_{sk}) + \frac{j_{ik}}{B_{ik}} - \frac{j_{sk}}{B_{sk}} = 0 \;\; i = 1, \dots, s-1; k = 1, \dots, m-1 \qquad \text{(S1)}$$

The constraint that for each discretization, the collective sum of all fluxes is zero is applied to derive the fluxes of component s for $k = 1, \dots, m-1$ discretization [3]:

$$j_{sk} + \sum_{i=1}^{s-1} j_{ik} = 0 \qquad \text{(S2)}$$

The interface velocity v is obtained by substituting equations 2 and 4 in equation 6 from the manuscript:

$$\frac{1}{\Omega}\left(g^{solid} - \sum_{i=1}^{s} x_i^{solid} \mu_{i,int}^{liq}\right) + \frac{v}{M} + \sum_{i=1}^{s} C_i K_i v = 0 \qquad \text{(S3)}$$

Here, $\mu_{i,int}^{liq}$ refers to μ_{i1}. Equations S1, S2 and S3 are simultaneously solved in MATLAB to obtain the values for j_{ik} ; $i = 1, .., s; k = 1, \dots m-1$ and v.

From the values of j_{ik} and v, the rate of change of mole fraction of Li_2O and SiO_2 in each discretization is derived as follows [3]:

$$\Delta_k \dot{x}_{ik} = \Omega(j_{ik-1} - j_{ik}) \; \mathrm{i} = 1, \dots, \mathrm{s} - 1; \; \mathrm{k} = 2, \dots, \mathrm{m} \qquad \text{(S4)}$$

$$\Delta_1 \dot{x}_{i1} = \Omega(\tilde{j}_{i1} - j_{i1}) \;\; \mathrm{i} = 1, \dots, \mathrm{s} - 1 \qquad \text{(S5)}$$

$$\tilde{j}_{i1} = -K_i v \;\; \mathrm{i} = 1, \dots, \mathrm{s} - 1 \qquad \text{(S6)}$$

Following equations S3, S4, S5 and S6, the interface position and the mole fraction for components $\mathrm{i} = 1, \dots, s-1$ are updated by the forward Euler methodology.

Finally, the constraint $\sum_{i=1}^{s} x_{i,k} = 1$ is applied to update $x_{s,k}$ for $\mathrm{k} = 1, \dots, \mathrm{m}$.

Reference

[1] Bale, C. W., Bélisle, E., Chartrand, P., Decterov, S. A., Eriksson, G., Gheribi, A. E., Hack, K., Jung, I.H., Kang, Y.B., Melançon, J., et al. (2016). FactSage thermochemical software and databases, 2010–2016. CALPHAD: Computer Coupling of Phase Diagrams and Thermochemistry, 54, 35-53.

[2] Li, H., Qiu, H., Schirmer, T., Goldmann, D., & Fischlschweiger, M. (2022). Tailoring Lithium Aluminate Phases Based on Thermodynamics for an Increased Recycling Efficiency of Li-Ion Batteries. *ACS ES&T Engineering*, *2*(10), 1883-1895.

[3] Svoboda, J., Gamsjäger, E., Fischer, F. D., & Fratzl, P. (2004). Application of the thermodynamic extremal principle to the diffusional phase transformations. *Acta materialia*, *52*(4), 959-967.
